# Dehydration and crystallization of amorphous calcium carbonate in solution and in air

**DOI:** 10.1038/ncomms4169

**Published:** 2014-01-28

**Authors:** Johannes Ihli, Wai Ching Wong, Elizabeth H. Noel, Yi-Yeoun Kim, Alexander N. Kulak, Hugo K. Christenson, Melinda J. Duer, Fiona C. Meldrum

**Affiliations:** 1 School of Chemistry, University of Leeds, Leeds LS2 9JT, UK; 2 Department of Chemistry, University of Cambridge, Lensfield Rd, Cambridge CB2 1EW, UK; 3 School of Physics and Astronomy, University of Leeds, Leeds LS2 9JT, UK

## Abstract

The mechanisms by which amorphous intermediates transform into crystalline materials are poorly understood. Currently, attracting enormous interest is the crystallization of amorphous calcium carbonate, a key intermediary in synthetic, biological and environmental systems. Here we attempt to unify many contrasting and apparently contradictory studies by investigating this process in detail. We show that amorphous calcium carbonate can dehydrate before crystallizing, both in solution and in air, while thermal analyses and solid-state nuclear magnetic resonance measurements reveal that its water is present in distinct environments. Loss of the final water fraction—comprising less than 15% of the total—then triggers crystallization. The high activation energy of this step suggests that it occurs by partial dissolution/recrystallization, mediated by surface water, and the majority of the particle then crystallizes by a solid-state transformation. Such mechanisms are likely to be widespread in solid-state reactions and their characterization will facilitate greater control over these processes.

## Abstract (web summary)

The crystallization of amorphous calcium carbonate is a widely studied process. Here, the authors probe the mechanism and show that transformation to calcite is preceded by dehydration, even in solution, and that loss of the final water fraction triggers crystallization.

## Graphical abstract

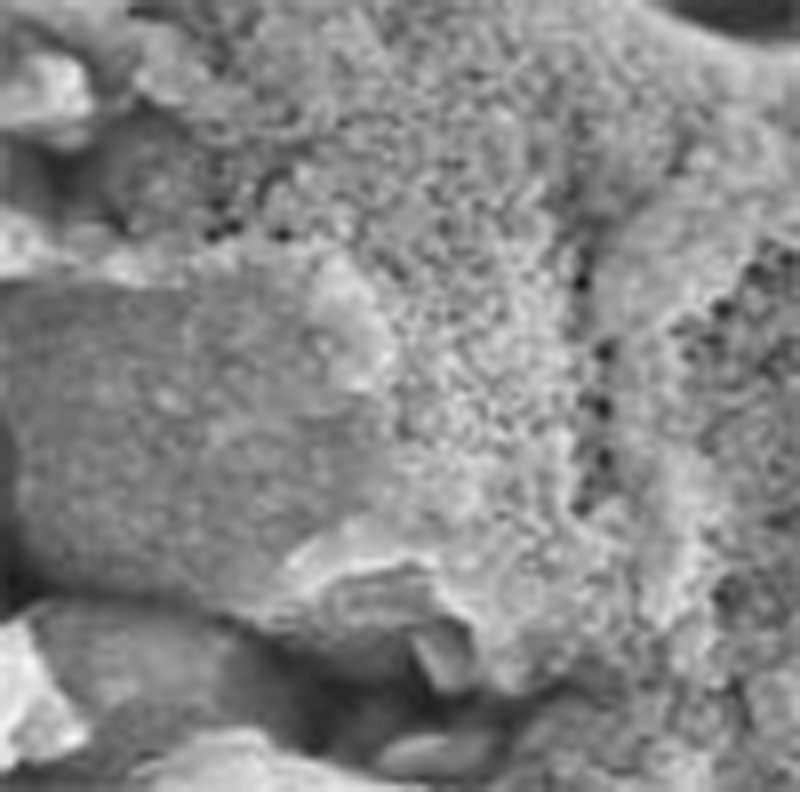

The recognition that hydrated amorphous precursor phases can play key roles in the formation of vertebrate and invertebrate biominerals has generated significant interest in these phases^1^,^2^,^3^,^4^. Providing organisms with a mouldable, space-filling starting material^5^, which can be delivered on demand for the rapid, yet controlled production of structurally and morphologically complex crystalline biominerals, this biogenic strategy offers a perfect candidate for biomimicry^6^. Focusing on amorphous calcium carbonate (ACC), it has proven possible to profit from ACC precursor phases to generate CaCO_3_ microlens arrays at the gas–liquid interface^7^, single crystals with complex forms via templating approaches^8^,^9^, thin films and fibres of calcite and vaterite in the presence of polyelectrolytes^2^,^10^,^11^ and inorganic/organic composites^12^,^13^. A range of synthetic approaches have also been used to control the crystallization of ACC. For example, the stability of ACC can be tuned using soluble inorganic and organic additives^14^,^15^,^16^ by association with insoluble matrices^17^,^18^ and by varying the particle size^19^. However, widespread exploitation of ACC in materials synthesis has hitherto been limited by the challenges of characterizing structure^14^,^20^,^21^, tuning stability^17^, controlling morphologies^6^ and determining crystallization mechanisms.

A significant contribution to our current understanding of ACC and its crystallization has of course come from the study of biominerals. Biogenic ACC can be classified as either stable or transient, where the stable form is hydrated (with approximate composition CaCO_3_:H_2_O) and the transient form anhydrous^1^. The best characterized transient system is that of the sea urchin embryo, in which tri-radiate, single-crystal calcite spicules form from ACC within a membrane-bound vacuole. The ACC is tightly bound by the membrane such that the system remains free of bulk water^4^,^22^,^23^,^24^, and under these conditions the initial hydrated ACC is observed to dehydrate to a more stable anhydrous ACC phase, before it subsequently crystallizes via a ‘solid-state’ mechanism^24^,^25^. While a comparable stepwise transformation has been observed when ACC is heated in air to drive off the water^15^,^26^,^27^,^28^,^29^, the mechanisms of these structural transformations are not yet well understood. By comparison, ACC typically crystallizes very rapidly in aqueous solution, such that characterization of this process has proven extremely challenging^30^,^31^,^32^,^33^,^34^,^35^.

This work therefore employs a bio-inspired strategy to address an intensively debated topic—the crystallization mechanism of ACC–by characterizing the transformation of synthetic ACC in aqueous environments. Encapsulation of ACC particles within porous silica shells provides an effective inorganic mimic of the spicule environment of the sea urchin, thereby sufficiently retarding the crystallization to allow characterization of the transformation. The mechanisms and structural changes that accompany ACC dehydration in air were also investigated in detail with ACC samples with well-defined water contents generated by annealing at different temperatures, and characterizing these using thermogravimetric analysis (TGA) and solid-state NMR. Comparison of both systems demonstrates that while identical dehydration processes can occur both in air and in solution, ACC crystallization at room temperature must be initiated by a local dissolution/reprecipitation, as mediated by water present on the particle surface or in the environment. The majority of the particle can then crystallize via a solid-state transformation.

## Results

### Crystallization of silica-coated ACC particles

**Figure 1:**
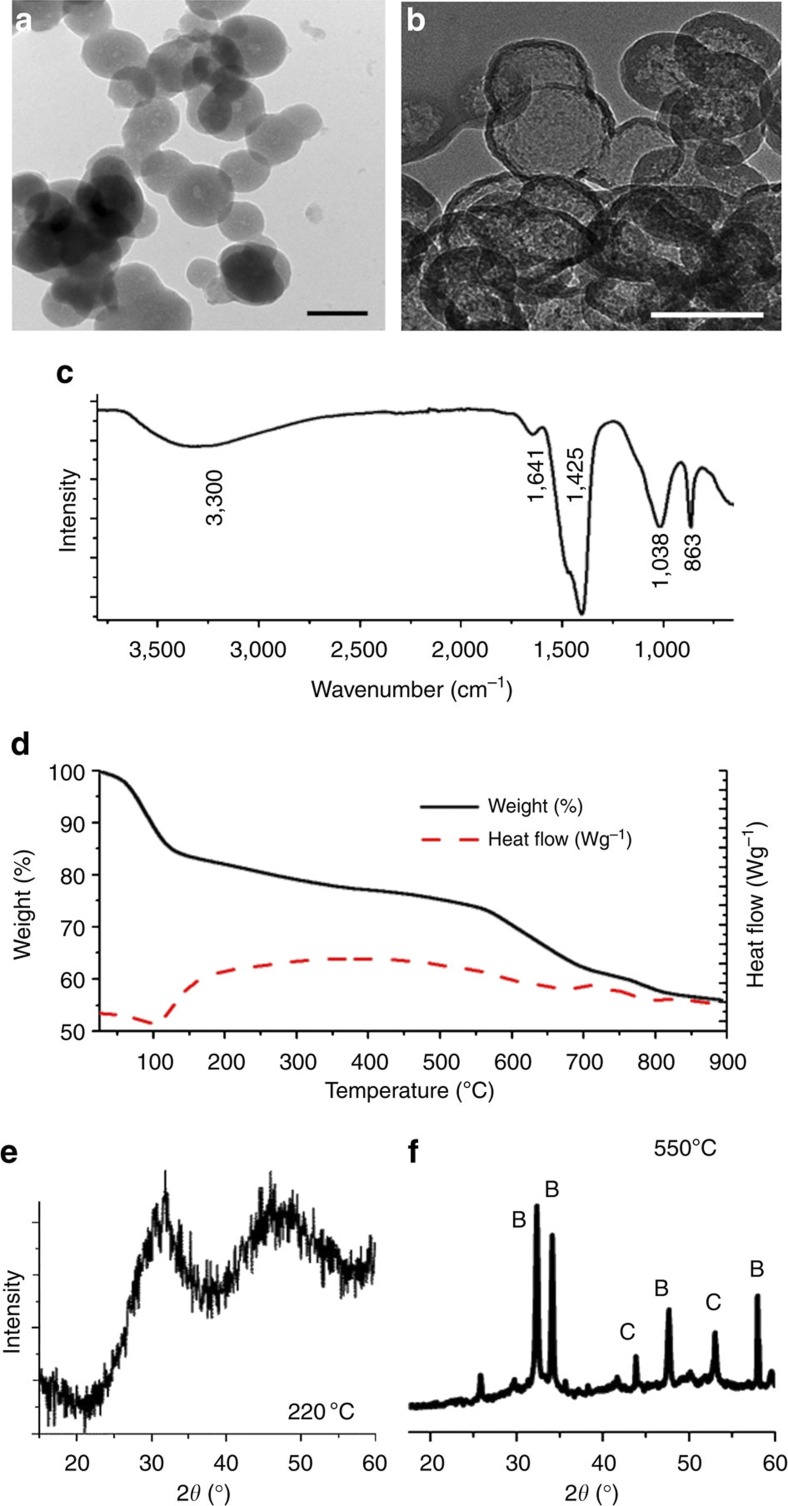
**Analysis of as-prepared silica-coated ACC particles ACC-SiO**_**2**_. TEM images of (**a**) ACC-SiO_2_ particles and (**b**) silica shells formed after dissolution of the ACC core—scale bar, 100 nm. (**c**) Infrared spectrum and (**d**) TGA of ACC-SiO_2_ particles, and PXRD spectra of ACC-SiO_2_ particles after heating to (**e**) 220 °C and (**f**) 550 °C. B, belite; C, calcite.

Transmission electron microscopy (TEM) confirmed that the silica-coated ACC particles^36^ (prepared by simple mixing of solutions of CaCl_2_ and Na_2_CO_3_/Na_2_SiO_3_) had diameters of ~100 nm (Fig. 1a), while subsequent leaching of the encapsulated ACC through incubation in 1 M HCl for 24 h demonstrated that a continuous ~5–10 nm thick silica shell forms around each ACC particle (Fig. 1b). Infrared spectra (Fig. 1c) showed bands characteristic of both ACC and amorphous silica, with peaks corresponding to the carbonate group appearing at 1,425 cm^−1^ (*ν*_3_), 1,075 cm^−1^ (ν_1_) and 863 cm^−1^ (*ν*_2_), due to water at 1,641 cm^−1^ and silica at 1,038 cm^−1^. Bands (*ν*_4_) at 747 and 714 cm^−1^, which are the characteristic of crystalline vaterite and calcite, respectively, were notably absent.

TGA of freshly precipitated ACC-SiO_2_ particles (Fig. 1d) showed an 18–20 wt% loss below 200 °C due to dehydration of the ACC and SiO_2_, and an additional 7–10 wt% loss between 200–550 °C, attributed to release of CO_2_ on reaction of the SiO_2_ shell with the CaCO_3_. Powder X-ray diffraction (PXRD) was also performed during *in situ* heating of the ACC-SiO_2_ particles, where this demonstrated transformation from ACC (Fig. 1e) to Ca_2_SiO_4_ (belite) and calcite over 400 °C (Fig. 1f). A gradual weight loss of 18–20 wt% above 550 °C was observed rather than the sharp transition typically observed on conversion of CaCO_3_ to CaO. TGA of the silica shells alone that is, after leaching out the ACC revealed that they comprise ~20 wt% water (Supplementary Fig. 1). With a 100-nm particle diameter, a 5-nm-thick silica shell, and ACC and hydrated silica (SiO_2_·H_2_O) densities of 1.62 g cm^−3^ (ref. 37) and ~1.9 g cm^−3^, respectively, the ACC-SiO_2_ particles have compositions of ~22 wt% SiO_2_·H_2_O and ~78 wt% CaCO_3_·H_2_O. Given that the unheated ACC-SiO_2_ particles comprise 20 wt% water, ~4–6 wt% H_2_O is associated with the SiO_2_ component and ~14–16 wt% with the ACC.

**Figure 2:**
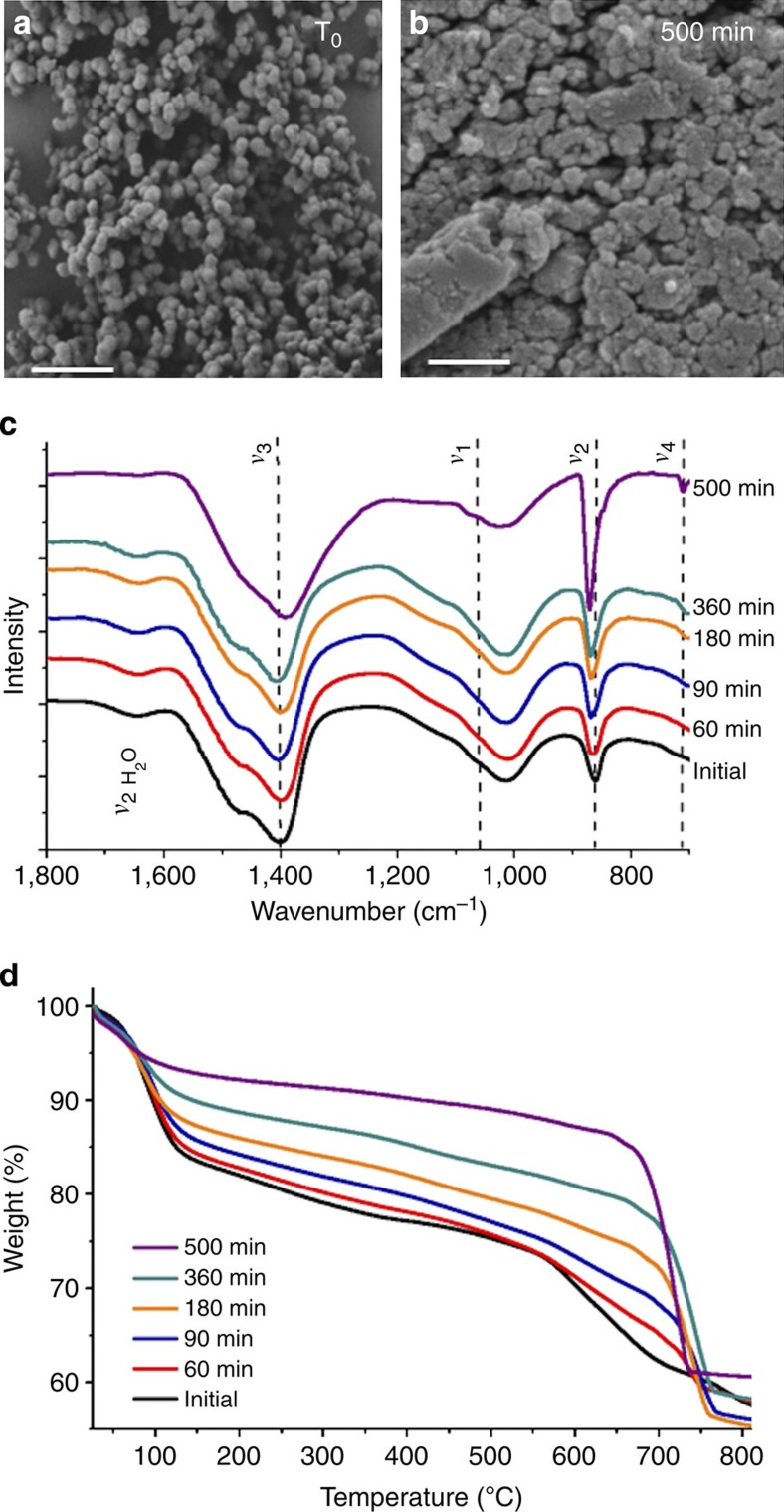
Dehydration of ACC-SiO_2_ particles on incubation in solution. SEM images of ACC-SiO_2_ particles after incubation in solution for (**a**) 0 min and (**b**) 500 min—scale bar, 500 nm. (**c**) Infrared spectra and (**d**) thermogravimetric analyses of ACC-SiO_2_ particles, showing the structural and compositional changes in the particles with incubation in solution.

The stability and crystallization in solution of ACC-SiO_2_ was investigated by resuspending 15 mg particles in 100 ml Milli-Q water and characterizing their structures and compositions with time (Fig. 2). Scanning electron microscopy (SEM) demonstrated the structural stability of the particles and showed that they aggregate during incubation (Fig. 2a,b) where infrared and TGA confirmed that this is accompanied by negligible change in the silica content of the particles (Fig. 2c,d and Supplementary Table 1). Addressing changes that occur in the ACC during their incubation in water, time-dependent infrared measurements (Fig. 2c) revealed a structural rearrangement, as was apparent from a narrowing of the *ν*_*3*_ band, a reduction in intensity of the *ν*_1_ absorption band and a shift in the *ν*_2_ band. Importantly, this was accompanied by dehydration of the ACC, which occurs before any evidence of crystalline phases is detected. The onset of crystallization occurs after ~8 h, as shown by the appearance of a characteristic calcite peak at 714 cm^−1^ (*ν*_4_).

TGA of ACC-SiO_2_ samples incubated in solution for different times clearly showed a decrease in the water-associated weight loss under 200 °C from 20 wt% to a constant 6 wt% (due to the SiO_2_·H_2_O phase, Fig. 2d). Also observed was the gradual appearance of a sharp CaCO_3_ to CaO transition above 550 °C (which is observed for uncoated ACC), and a reduction in the weight loss in the intermediate range (200–500 °C) for longer incubation times. Both of these phenomena demonstrate reduced calcium silicate formation in ACC samples with greater degrees of dehydration. This can be explained by the fact that co-precipitation of ACC in the presence of silicate also results in the occlusion of the silicate ions within the ACC, which results in an increase in its thermal stability^38^. During dehydration/restructuring of the ACC, silicate ions are likely to be expelled, resulting in reduced calcium silicate formation and possibly also the aggregation behaviour observed by SEM. Confirmation that the presence of silicate ions within the ACC does not change the pathway by which it crystallizes was obtained by monitoring the crystallization in water of ACC particles, which were precipitated in the absence of silicate, and then coated with a silica shell. While this method is less satisfactory as it never succeeds in completely coating every ACC particle present, the data obtained clearly demonstrate that the pure ACC particles also dehydrate before recrystallization (Supplementary Fig. 2).

We also explored the combined effects of encapsulation and stabilizing soluble additives with this system. It is well recognized that soluble macromolecules and ions, such as Mg^2+^, silica, sulphate and phosphate at moderate concentrations (<<[Ca^2+^]), contribute to the extended lifetime of biogenic ACC^22^,^39^,^40^. However, this alone cannot provide the stability observed for biogenic ACC^15^, strongly suggesting that the environment of the ACC within an organism also makes a significant contribution to its stability^39^,^41^. ACC was therefore precipitated in the presence of the crystallization inhibitor aspartic acid, and its crystallization was investigated. ACC-Asp-SiO_2_ particles crystallized by an identical pathway (dehydration followed by crystallization) where a small band at ≈700 cm^−1^ corresponding to crystalline CaCO_3_ was observed in infrared spectra after 18 h. This compares with the appearance of an equivalent peak at 8 h for ACC-SiO_2_ and under 1 h for uncoated ACC-Asp^15^ (Supplementary Fig. 3). The soluble additive and confinement therefore appear to act synergistically in retarding ACC crystallization.

### Crystallization of lipid bilayer-coated ACC particles

**Figure 3:**
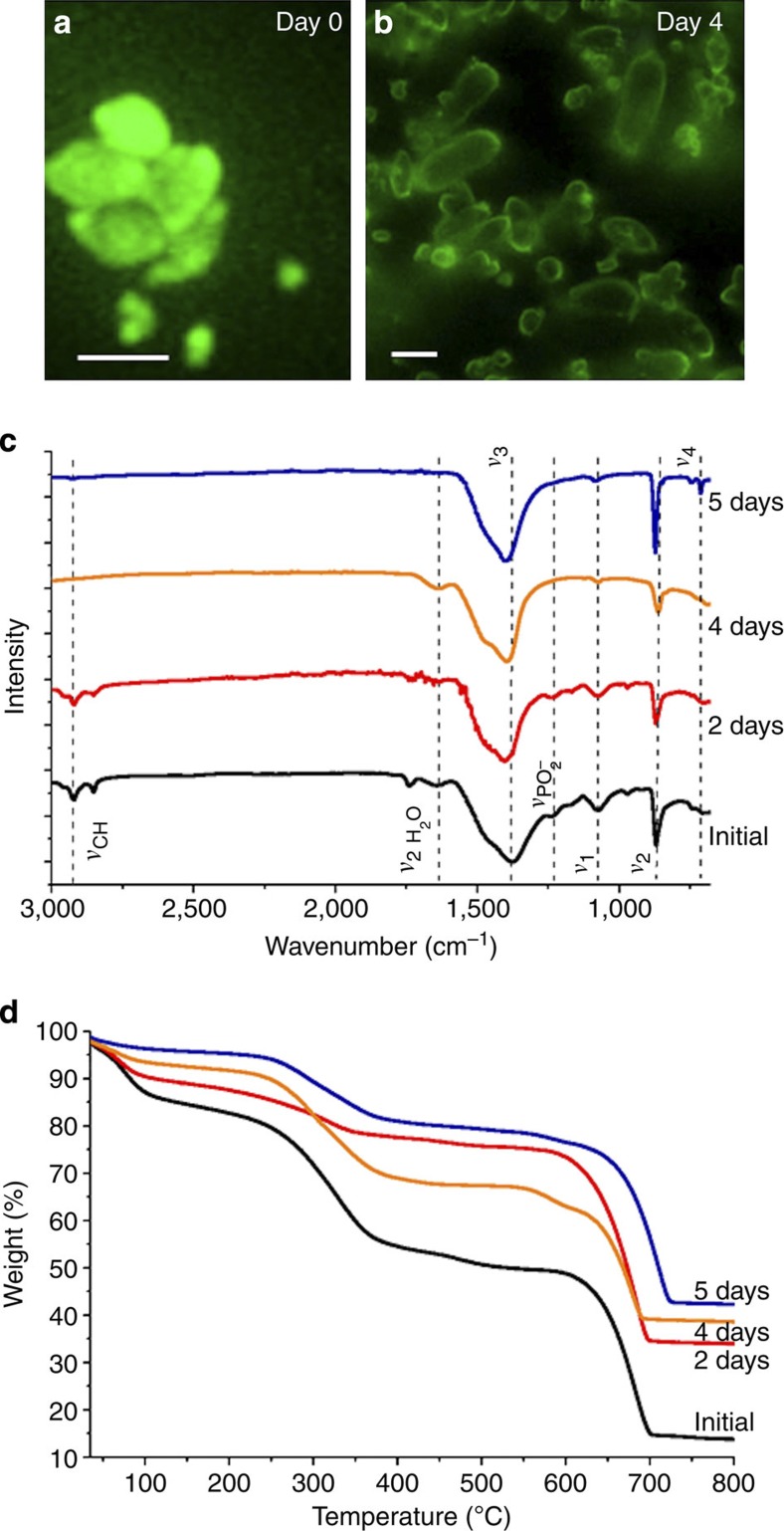
Characterization and dehydration of ACC particles coated with lipid bilayers. Laser scanning confocal microscopy images of aggregates as prepared (**a**)—scale bar, 1 μm and after 4 days (**b**) incubation in solution—scale bar, 4 μm, (**c**) Infrared spectra and (**d**) thermogravimetric analyses of ACC particles coated with lipid bilayers after certain incubation periods in solution.

Having demonstrated that encapsulation of ACC within a porous silica shell reduces the rate of ACC crystallization in solution, we extended our approach to explore whether ACC encapsulation within a lipid membrane—as in biological systems^4^,^23^—may act in an analogous way. ACC particles were coated with phosphatidylcholine-dihexadecyl phosphate (DHP) membranes using standard methods^42^, and their stability in Milli-Q water was investigated by isolating and characterizing the coated particles at different times. Laser scanning confocal microscopy, made possible by addition of a fluorescent phosphocholine (PC) molecule to the lipid mixture, demonstrated that the ACC particles were coated by lipid membranes and that they agglomerated with time (Fig. 3a,b). Notably, structural changes in the ACC comparable to those seen during the transformation of ACC-SiO_2_ were observed on incubation in solution, as shown by a reduction in intensity of the *ν*_1_ absorption band and a shift in the *ν*_2_ band. There was also a reduction in the intensity of the bands associated with the PC/DHP membrane^43^, (2,923 cm^−1^ (*ν*_CH_) and 1,234 cm^−1^ (*ν*_PO2_^−^)). No bands at 714 cm^−1^ (calcite) or 747 cm^−1^ (vaterite) were detected even after 4 days of incubation (Fig. 3c).

TGA of freshly prepared samples showed a weight loss due to lipid decomposition of ~30 wt% between 230 and 530 °C (Fig. 3d). This compares with ~20 wt% loss, estimated for 100 nm ACC spheres coating with single bilayers^44^, which suggests the presence of multilamellar coatings or additional vesicles. Importantly, the TGA analysis also demonstrated that the coated ACC particles underwent a very slow dehydration during incubation in solution, shown by the loss of water below 230 °C. Indeed, the water content decreased from an initial ~18–20 wt% to 10–13 wt% after 2 days, although the particles were still ACC, as judged by infrared spectroscopy. The mass loss associated with decomposition of organic materials that occurs at 230–530 °C also decreases from ~30 to 16 wt% over 2–4-day incubation. The data therefore indicate that the lipid coating of the ACC particles is lost/undergoes reorganization with time in solution, precluding investigation of extended periods leading to crystallization. However, the data clearly show that a lipid membrane can effectively stabilize ACC and that the ACC dehydrates before crystallization.

### Characterization of ACC with different hydration levels

**Figure 4:**
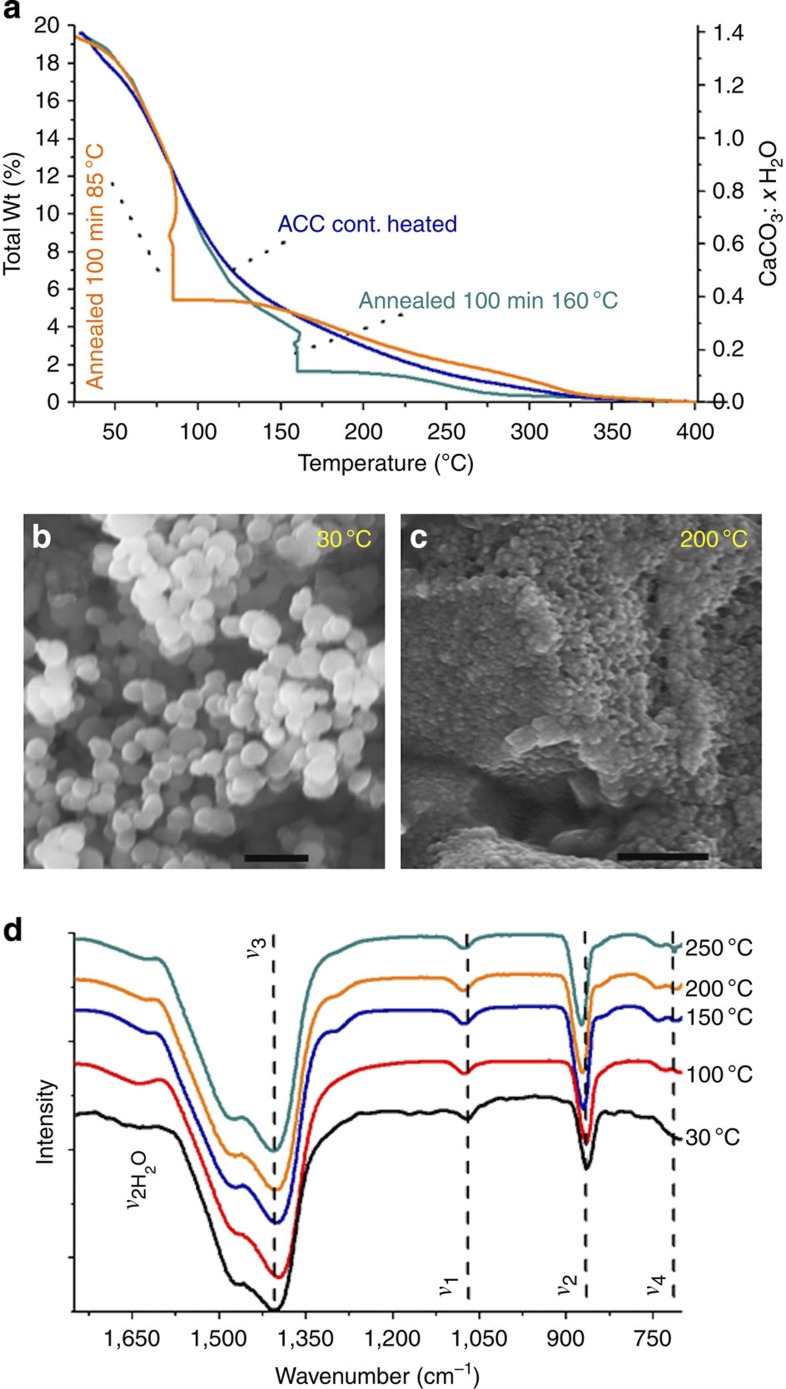
Crystallization of uncoated ACC particles. (**a**) Total weight percent water (Total wt%) and corresponding molecular composition (CaCO_3_: *x*H_2_O) from TGA from 25 to 400 °C), electron micrographs with a scale bar, 200 nm after isothermal annealing at 30 °C (**b**) and 200 °C (**c**), (**d**) infrared spectra of ACC particles after a heating procedure involving a ramp (15 °C min^−1^), followed by isothermal annealing (100 min) and a second ramp (15 °C min^−1^) all under a 100-ml min^−1^ N_2_ flow).

Detailed studies of the transformation from hydrated ACC to anhydrous ACC to crystalline calcite were then performed by annealing ACC samples at specific temperatures. TGA showed that ACC precipitated from the combination of 1 M CaCl_2_ with 1 M (NH_4_)_2_CO_3_ contained 20±1 wt% water, with 15±1 wt% water structurally associated with the ACC, which is consistent with the commonly reported molecular composition of ~CaCO_3_:H_2_O. ACC samples were then heated to, and isothermally annealed at, specific temperatures (between 25 and 220 °C in 5 °C steps) under a N_2_ stream, until the weight of each sample had stabilized. TGA/differential scanning calorimetry (DSC) was subsequently used to determine the amount of water lost at each temperature, the water fraction remaining and the crystallization onset temperature. Figure 4a shows representative TGA spectra that clearly demonstrate that ACC can be systematically dehydrated by application of defined heating cycles (a full range of curves is given in Supplementary Fig. 4). SEM showed that the 50-nm ACC particles aggregated during heating (Fig. 4b,c), while infrared spectroscopy (Fig. 4d) confirmed that the ACC remained amorphous after each isothermal annealing. Dehydration was accompanied by a structural rearrangement in the ACC, which on careful scrutiny shows up as a narrowing of the *ν*_3_ band, a reduction in intensity of the *ν*_1_ absorption band and a slight shift in the *ν*_2_ band to higher frequencies. Notably, these spectral changes are identical to those observed for ACC samples undergoing crystallization in solution (Fig. 2c). DSC showed that crystallization only occurred at temperatures above 290 °C, regardless of whether samples were continuously heated or whether they had been annealed at different temperatures. Crystallization activation energies of ~100 kJ mol^−1^ were derived in all cases using standard methods (Supplementary Figs 4 and 5)^45^ and can be compared with reported values of 73 kJ mol^−1^ (ref. 31) or 151–304 kJ mol^−1^ (ref. 28), depending on preparation conditions.

The activation energies associated with liberation of different water fractions were derived as averages of at least six isothermal measurements using equation (1) (ref. 46). Here *α* (as defined in equation (2)) represents the degree of dehydration, *A* is a pre-exponential factor, f(*α*) describes the reaction model and *W*_max_, *W*_min_ and *W*_t_ are the fractions of H_2_O present at the beginning, end and time *t* during an isothermal dehydration event.


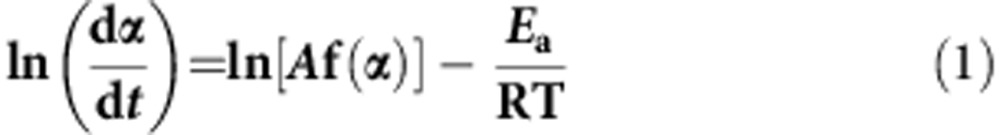



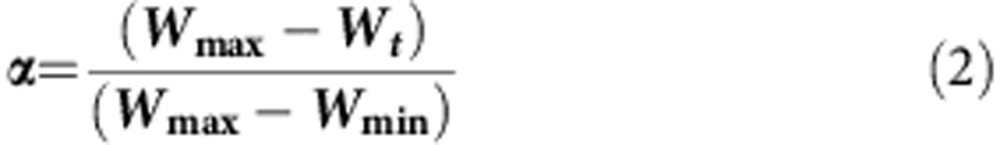


**Figure 5:**
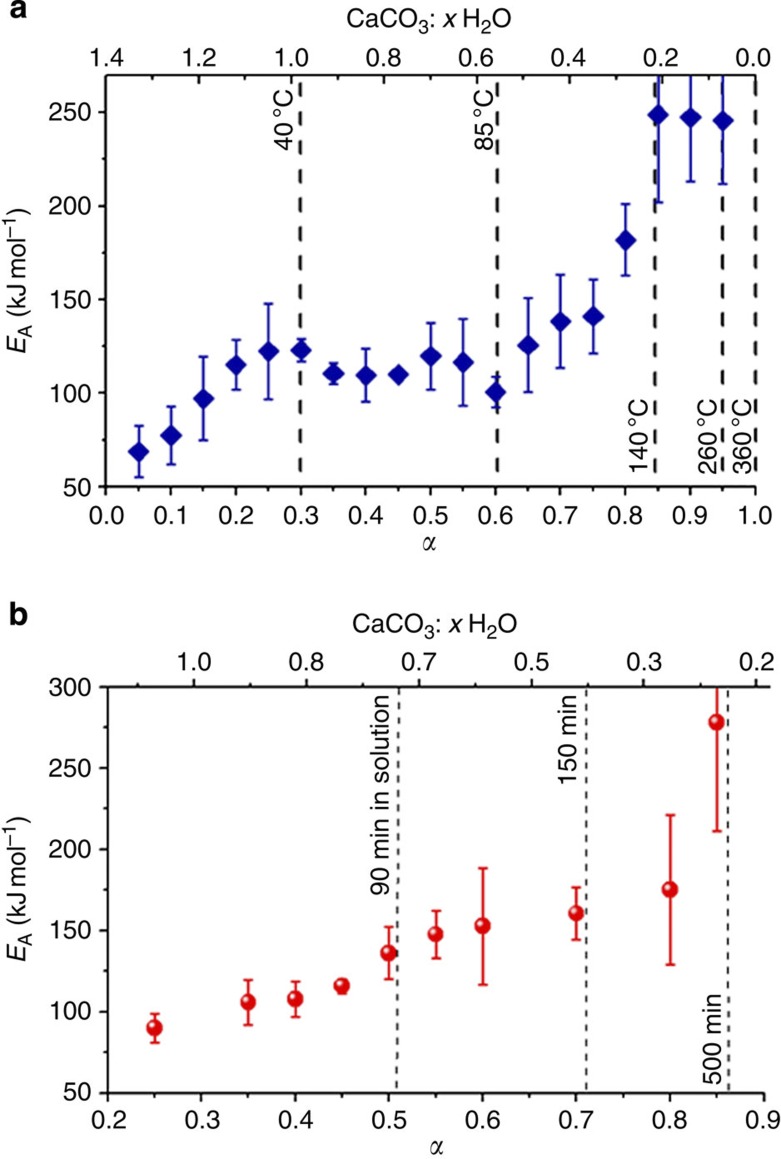
Activation energies of dehydration. The activation energies (*E*_a_) as a function of the degree of dehydration (*α*)/molecular composition of ACC CaCO_3_: *x*H_2_O (all water) for uncoated ACC particles (**a**) and ACC-SiO_2_ particles (**b**). The error bars are s.d.

Plots of these activation energies are given in Fig. 5 and show a general increase in the activation energy with increasing dehydration. Further, they indicate the existence of three apparent dehydration regimes. The first shows an increase in *E*_a_ up to *α*~0.2–0.3, which corresponds to the loss of the surface water, while the second corresponds to a plateau regime from 40 to ~85 °C (0.3≤*α*≤0.6). The *E*_a_ then increases to a regime from 140–260 °C (0.85≤*α*≤1), which is characterized by high activation energies of ~245 kJ mol^−1^ (Fig. 5a). Estimates of the weight loss and activation energies (*E*_a_) of each of the dehydration regimes are summarized in Table 1. The dehydration of the silica-coated ACC particles in air was also similarly assessed to determine the influence of the silica shell on ACC dehydration. The activation energies also increased as dehydration progressed, although no well-defined plateau regions were observed. The derived activation energies were somewhat higher than for the uncoated ACC in air, demonstrating that the silica coating can retard ACC crystallization by providing a barrier to water loss (Fig. 5b).

### Mechanism of dehydration of ACC

**Figure 6:**
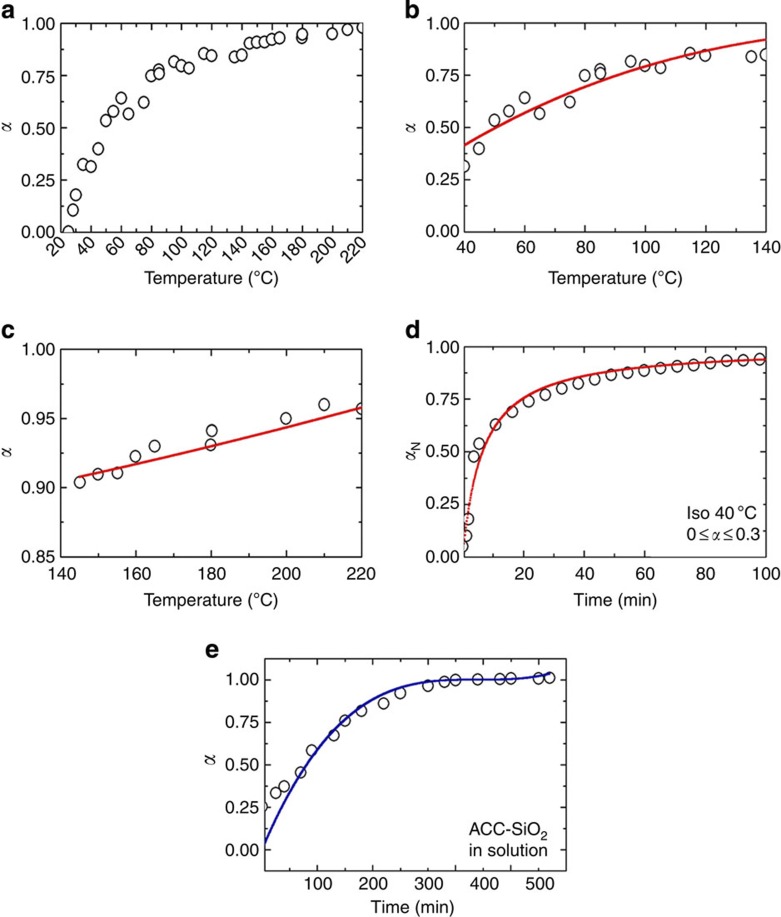
Analysis of the progress of dehydration. Dehydration curves of ACC and ACC-SiO_2_ particles as a function of temperature (*T*) or time (*t*), together with best fits to the common solid-state reaction models described in Table 1. (**a**) Dehydration of uncoated ACC on heating in air is shown over the range 20–220 °C. Panels **b** and **c** show specific ranges of this dehydration process where **b** shows the range 40–140 °C, and is fitted by a geometric contraction and **c** shows the range 140–220 °C, which is fitted by a second-order nucleation model. Panel **d** shows the dehydration of the same sample under isothermal annealing at 40 °C. (**e**) Dehydration as a function of time for ACC-SiO_2_ particles on incubation in solution at 25 °C. *α* is the total fraction of water (as in Fig. 5), while *α*_N_ refers only to the fraction of water lost over each particular range (from 0 at the start to 1 at the end).

**Figure 7:**
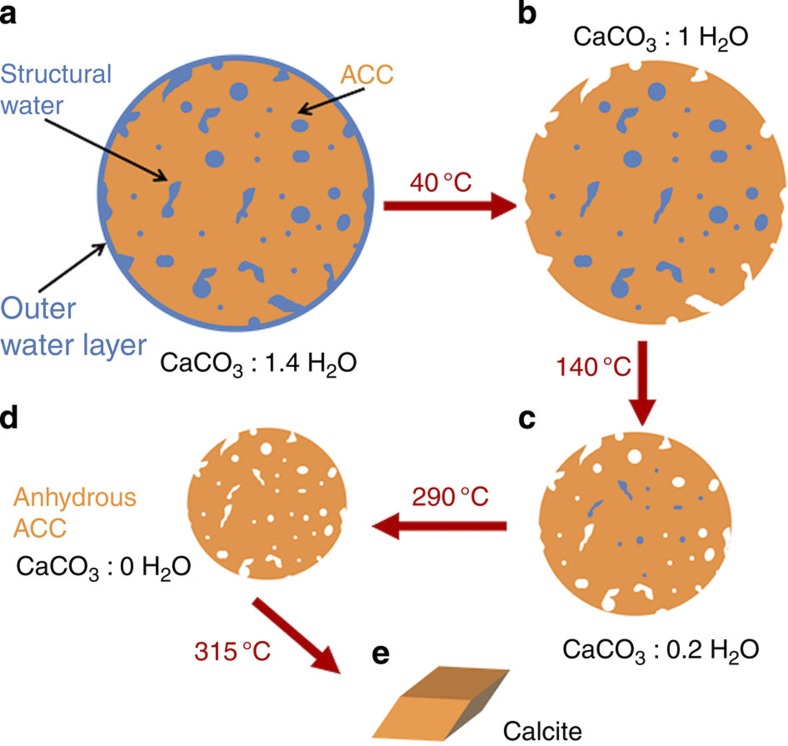
Schematic of stages of dehydration. On going from **a** to **b**, surface-bound water is lost, during **b** to **c** water is lost from the interior of the ACC and the ACC particle shrinks. On going from **c** to **d**, the most deeply located water is expelled and on going from **d** to **e**, crystallization to calcite occurs.

Plots of the gradual dehydration of ACC in air over the range 25–220 °C were derived using *α* values obtained at the end of each annealing period (Fig. 6). The rate of dehydration decreases at higher temperatures, demonstrating that it becomes increasingly difficult to remove water as the limit of anhydrous ACC is reached, in keeping with the activation energy measurements (Fig. 5). The dehydration curve also provides further insight into the mechanism of dehydration of ACC in air by considering fits to common solid-state reaction models (f(*α*)) (ref. 47) are presented in Table 1. While the validity of such analysis has been widely debated because of the mathematical interdependence between activation energy, pre-exponential factor and chosen model^48^,^49^, this method provides some insight into the dehydration mechanisms that may operate. The full dehydration curve (Fig. 6a) is best described by a geometric contraction model, in which the reaction rapidly initiates on the particle surface and then proceeds towards its centre. The intermediate temperature range (40–140 °C), which represents 65% of the total water fraction, can also be described by the same model (Fig. 6b). In both cases, a contracting sphere provided a slightly better fit than a contracting cylinder, with *R*^2^ values of 0.92 and 0.89, respectively, as compared with 0.90 and 0.85. The final dehydration at 140–220 °C, which represents less than 15 wt% of the initial water content, is in contrast best described by a second-order nucleation model (Fig. 6c). Removal of the last water is therefore not diffusion limited but is determined by the barriers to water release. The dehydration regime from 0≤*α*≤0.3 (~40 °C) is best described by an isothermal process following a second-order rate equation, as is consistent with loss of surface water and common adsorption isotherms (Fig. 6d). The dehydration of the ACC-SiO_2_ particles in solution as a function of time (Fig. 6e) showed that the overall behaviour from 0.3≤*α*≤1 obeys an identical three-dimensional model (contracting sphere, *R*^2^=0.94) as for the dehydration of uncoated ACC in air. A schematic of the dehydration mechanism according to the overall contraction model is given in Fig. 7. These analyses therefore strongly support the existence of the distinct dehydration regimes identified using the derived activation energies.

### NMR analysis

**Figure 8:**
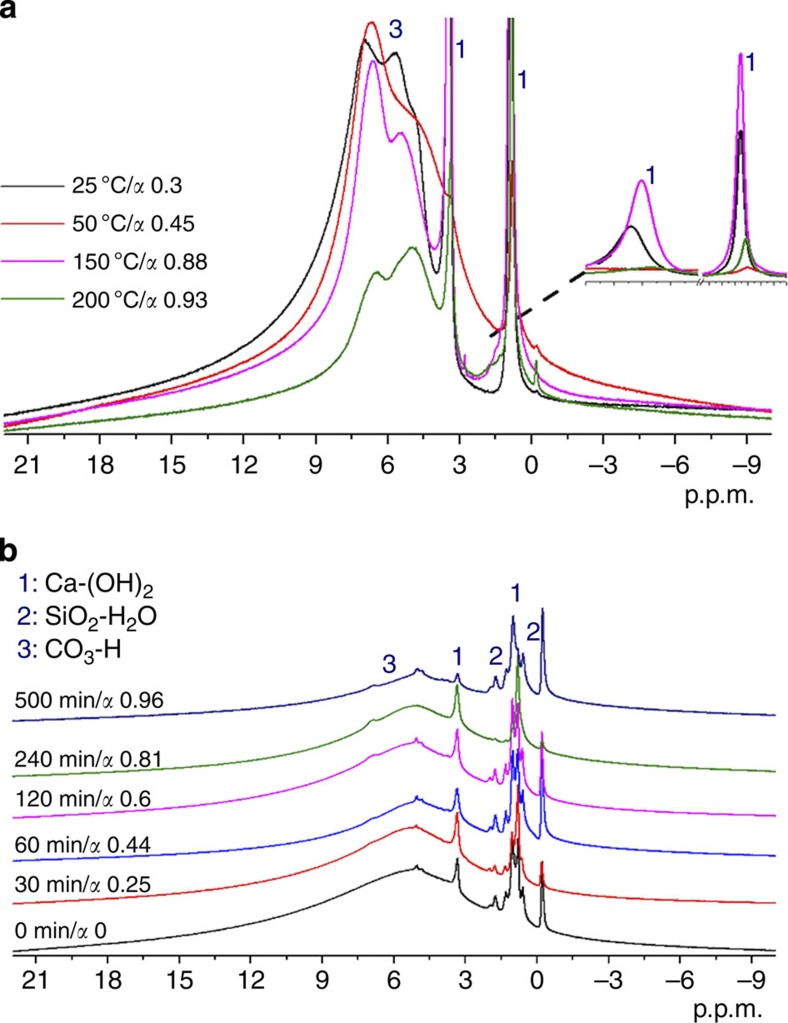
Solid-state NMR analysis. ^1^H solid-state NMR spectra of (**a**) uncoated ACC particles and (**b**) ACC-SiO_2_ particles with different levels of dehydration, where the ACC-SiO_2_ particles were dehydrated by incubation in solution and the uncoated ACC particles were dehydrated through isothermal heating.

Further insight into the nature of the water environments in hydrated ACC was gained from ^1^H solid-state NMR (SSNMR) measurements of ACC samples that had been isothermally annealed to different levels of dehydration. Analysis of a fully hydrated sample demonstrated the presence of five different proton environments, namely, a rigid structural phase associated with Ca^2+^ (two types of OH^−^ at 0.9 and 3.4 p.p.m.), two partially mobile phases due to H_2_O (4.9, 5.7 p.p.m.) and a signal due to CO_3_^2−^ (H^+^) framework components (7 p.p.m.; Fig. 8)^50^. As dehydration progressed, there was little change in the OH^−^ signal, while the ^1^H signal from H_2_O and HCO_3_^−^ decreased. Heating of the samples resulted in coalescence of the ^1^H signals giving a broader signal centred at ~5.2–5.5 p.p.m., that is, a weighted average of the 4.9 and 5.7 p.p.m. signals. This is due to exchange of protons between the two environments, showing that they are in physical contact. The ^1^H signal from HCO_3_^2−^ shifts downfield (~6.7 p.p.m.) when the dehydration temperature is increased, suggesting that the ^1^H in these sites also exchange with water ^1^H, so that its chemical shift becomes a weighted average of that for the HCO_3_^−^ site (~7 p.p.m.) and the water sites (4.9, 5.7 p.p.m.). ACC-SiO_2_ particles with different water contents were also characterized, where these were generated on incubation in solution for different times. Again, as-prepared samples showed the presence of different proton environments within the ACC, along with signals originating in the hydrated SiO_2_ shell^51^.

## Discussion

By employing a bio-inspired strategy, where encapsulation of ACC particles within silica shells retards crystallization, we here show that in common with biomineralization processes and the transformation of ACC in air, synthetic ACC also dehydrates at room temperature in an aqueous environment. This is driven by the generation of a more stable, low water content ACC phase^26^. Characterization of this dehydration process revealed a strong dependence of the activation energy required to remove water on the degree of dehydration. The activation energies required to remove the first water fractions (up to 0.3 H_2_O) are close to the hydration energy of calcite faces in a humid atmosphere^52^, as expected if the first stage of dehydration removes more accessible water of hydration. Note that this is far more than monolayer coverage of water on the outer surface of the ACC particles, and undoubtedly includes water condensed around the contact points of adjacent particles as well as some more deeply located water. The higher activation energies measured for the remaining fractions may reflect the increasingly hindered escape of water molecules in low humidity environments^53^.

Although it has been noted that a mechanistic interpretation of the magnitudes of activation energies for solid-state reactions is well-nigh impossible^54^, the results are in good agreement with modelling studies of ACC dehydration. These predict an increase in hydration energy with increasing degrees of dehydration owing to the formation of a stronger hydrogen-bond network surrounding neighbouring Ca^2+^ and CO_3_^2−^ ions^55^. This may also be associated with the observed structural reorganization of the ACC during heating. A model of ACC dehydration has also been predicted by combined computer simulations and structural studies of synthetic ACC (CaCO_3_·H_2_O)^56^, which suggested that the water molecules in hydrated ACC are located, along with carbonate ions, within a network of nanoporous channels in a Ca^2+^-rich framework. These channels could therefore provide a conduit for loss of water during dehydration, where this process would also be accompanied by a structural rearrangement in which CO_3_^2−^ ions relocate from the channels into the calcium framework. Again, channels may close at higher levels of dehydration. The results obtained in this work are consistent with either model.

Our study also provides insight into the crystallization process itself. Considering first dry ACC, crystallization is only observed at temperatures of ~300 °C, where this is triggered by/coincides with the loss of the final water fraction. With no water present, the transformation of anhydrous ACC to calcite must proceed by a solid-state transformation. Indeed, our experiments show that the final dehydration step is associated with a very high activation energy of ~245 kJ mol^–1^. Activation energies of dehydration of crystalline solids vary widely but are typically of the order of 100 kJ mol^−1^ (ref. 54). That ACC crystallizes very rapidly in solution at room temperature indicates that this must occur by an alternative mechanism with a lower-energy barrier. Indeed, when isolated, ACC only shows extended stability when washed with solvents such as ethanol, which can substitute for much of the surface water^57^. Even then, the rate of crystallization is dependent on the ambient humidity. Further, ACC that has no surface-bound water (as prepared by freeze drying) is extremely stable, only crystallizing under normal levels of humidity after 6 weeks^58^.

These data therefore strongly suggest that while ACC can certainly dehydrate at room temperature, the free energy barrier to nucleation is such that formation of the first crystalline phase can only occur via a partial dissolution/reprecipitation. We emphasize that we are not proposing that the ACC fully dissolves and then reprecipitates. Instead, this would appear most probably to occur within a domain on the surface of an ACC particle, or could happen within an ACC particle containing pockets of entrapped water. Crystallization of the entire ACC particle can then occur by a solid-state transformation, which has also been termed secondary nucleation^59^, where the presence of the crystal nucleus induces structural changes in the adjacent ACC^24^; the low water content of the ACC precludes local dissolution/crystallization. Such a transformation is supported by the structural changes that accompany dehydration of the ACC. The transformation mechanism of ACC at room temperature is therefore defined by a balance between the rates of dehydration and dissolution/reprecipitation. ACC with surface water would be expected to transform via the solid-state mechanism mentioned above, while a full dissolution/reprecipitation mechanism would be anticipated for ACC in bulk solution.

These mechanisms are also consistent with data presented in the literature. *In situ* small angle X-ray scattering/wide angle X-ray scattering studies of CaCO_3_ precipitation in concentrated (1 M) solutions have suggested that initial dehydration or reorganization of ACC, followed by direct transformation to vaterite, occurs at early reaction times, before changing to a dissolution-reprecipitation mechanism^30^,^31^. Evidence for a direct transformation also comes from cryo-TEM studies of ACC transformation into vaterite, which revealed the development of nuclei within the ACC particles^32^. Further support for nucleation of the new crystal phase within ACC comes from observations that ACC typically aggregates before direct transformation into a crystal^60^ that ACC particles crystallize more slowly in small volumes with few particles present,^61^,^62^ and that small ACC particles show greater stability^19^. Once initial nuclei of vaterite or calcite are established, subsequent growth occurs via dissolution of the surrounding ACC, as shown by depletion zones around crystal nuclei^17^,^63^, through measurements of the changes in the solution composition on crystallization^33^,^34^ and by simultaneous small angle X-ray scattering/wide angle X-ray scattering studies of CaCO_3_ precipitation^30^,^31^,^35^.

Looking beyond CaCO_3_, our results are also relevant to many other natural or synthetic transformations, such as amorphous titania to anatase or rutile, or ferrihydrite to goethite or haematite. These are hydrated, metastable, amorphous or nanocrystalline phases transforming after an initial dehydration, either thermally or in aqueous solution^64^. Similarly, a dissolution-recrystallization mechanism or a solid-state transformation have been proposed for the transformation of ferrihydrite, to haematite or goethite^65^. However, it is generally agreed that haematite formation involves an ‘internal’ dehydration (about a 25% weight loss) followed by crystallization, possibly by a topotactic route. Further examples are the dehydration of crystal hydrates, which often proceeds via an amorphous phase, as dehydration often destroys a crystal lattice^54^. Just as with ACC, the precise mechanisms of these transformations are still being vigorously debated.

In conclusion, our data provide insight into the mechanisms of transformation of ACC to crystalline polymorphs in biological, environmental and synthetic systems. Thermal analysis and SSNMR demonstrated that ACC undergoes parallel dehydration and structural changes both in solution and in air where these processes enable a subsequent ‘solid-state’ transformation. The water in ACC exists in different environments and it is the loss of the final component that triggers crystallization. This step is associated with a high free energy barrier such that at room temperature the first crystal nucleus can only form via a dissolution/reprecipitation mechanism mediated by water present on particle surfaces or in solution. The majority of the structural water present within hydrated ACC is therefore of little importance to its stability, but plays a key role in the initial precipitation of ACC, lowering the energy barrier towards the formation of this hydrated phase compared with the anhydrous crystalline polymorphs. Through application of a bio-inspired strategy, we also show that confinement stabilizes ACC, most probably by creating a barrier to water diffusion, by retarding dissolution/reprecipitation-based nucleation and by limiting ACC aggregation. This effect is enhanced in the presence of crystallization inhibitors, suggesting that nature employs both biomacromolecules and confinement to tailor the stability of ACC in organisms. CaCO_3_ is not unique in this multi-step crystallization pathway, and the mechanisms observed are likely to provide new insight into the formation of many common natural and synthetic materials.

## Methods

### Materials and general preparative methods

Analytical grade (NH_4_)_2_CO_3_, CaCl_2_·2H_2_O and L-aspartic acid sodium salt monohydrate were purchased from Sigma-Aldrich and used as received. Na_2_SiO_3_ solution (1.35 g cm^−3^) was from Merck Chemicals, and aqueous stock solutions were prepared using Milli-Q water, 18.2 MΩcm. Stock solutions of L-α PC (1,2-dipalmitoyl-sn-glycero-3-phosphatidylcholine, >99%, Sigma) were prepared in HPLC-grade chloroform. Glassware used to prepare solutions was soaked overnight in 10% w/v NaOH, followed by rinsing with dilute HCl and finally washing with Milli-Q water. Glass slides and crystallizing dishes were placed overnight in Piranha solution (70:30 wt% H_2_SO_4_: H_2_O_2_) and then washed copiously with Milli-Q water before drying with acetone.

### Preparation of ACC

Except where otherwise noted, ACC was produced by combining equal volumes (0.5–1.5 ml) of 1 M (NH_4_)_2_CO_3_ (pH 9.15) with 1 M CaCl_2_ (pH ~6.8) at 4 °C, and the ACC precipitate was immediately filtered through a 0.45-μm Isopore GTTP membrane filter (Millipore) before washing with ethanol, and drying over silica gel for 1 h.

### Synthesis of ACC-silica particles and hollow silica shells

ACC particles encapsulated in silica shells were synthesized following Kellermeier *et al*.^36^ In brief, 125 ml of 10 mM CaCl_2_·2H_2_O was mixed with 125 ml of 10 mM Na_2_CO_3_/6 mM Na_2_SiO_3_ solution, and the precipitates generated were incubated in the reaction solution for 10 min to allow formation of a silica shell. The solutions were then filtered using a 0.45-μm Isopore GTTP membrane filter (Millipore) and washed with ethanol before being left to dry. The dried ACC-silica particles (~15 mg) were subsequently dispersed in 100 ml of Milli-Q water and aliquots were removed at different times to investigate the crystallization mechanisms. Confirmation of encapsulation of ACC was obtained by leaching the ACC from the silica shell by immersing ~500 mg of ACC-SiO_2_ particles in 1 M HCl (50 ml) for 24 h.

### Dehydration of aspartic acid-stabilized ACC-silica particles

Silica-coated ACC particles stabilized with Asp were synthesized by combining equal volumes of 10 mM Na_2_CO_3_/6 mM Na_2_SiO_3_ and 10 mM CaCl_2_·2H_2_O/5 mM aspartic acid. The onset time of crystallization of these particles was then compared with that of pure ACC-silica particles, prepared as above.

### Synthesis of pure ACC particles coated with silica shells

ACC was also synthesized in the absence of silica and was then coated with a silica shell. This method enables characterization of the transformation of ACC in the absence of occluded silica but suffers from the limitation that a fraction of the original ACC particles are not completely coated, and therefore crystallize rapidly. ACC was prepared by direct combination of 0.5 ml of 20 mM CaCl_2_·2H_2_O with 0.5 ml of 20 mM Na_2_CO_3_. Post-deposition of silica was then achieved by the delayed addition (4 s after preparing the ACC) of 1 ml of 12 mM Na_2_SiO_3_. The transformation of these silica-coated ACC particles in water was then studied using infrared and TGA as described for ACC containing silica.

### Synthesis of ACC particles coated with lipid bilayers

Precipitated and dried ACC particles were coated with bilayers of L-α PC and DHP according to the method of Bugni^42^. ACC (5–25 mg) was dispersed in 1 ml of ethanol and were briefly sonicated (1–2 s), before depositing them on a glass slide and leaving to dry at 40 °C. Approximately 0.2 ml of a lipid stock solution (100 mg PC and 10 mg DHP per ml chloroform) was then applied dropwise to the ACC film, before rapidly evaporating the solvent under nitrogen. Subsequently, the resulting ACC bilayer aggregates were placed in 100 ml of Milli-Q Water and were gently agitated to displace them from the glass support. The transformation mechanism of the ACC bilayer particles was investigated by removing and analysing aliquots with time. The lipid bilayer coating on the ACC was confirmed using confocal fluorescence microscopy where particles were coated using a lipid stock solution containing PC labelled with a fluorescent group (1 wt% NBD-labelled PC (1-oleoyl-2-[12-[(7-nitro-2-1.3-benzoxadiazol-4-yl)amino]dodecanoyl]-sn-glycero-3-phosphocholine) (Avanti Polar Lipids).

### Preparation of ACC with different hydration levels

ACC containing different amounts of structural water was obtained by the simple heating and subsequent isothermal storage of ACC particles prepared in the standard manner. Samples were heated in a nitrogen atmosphere using a TA Instruments STD Q600 at a rate of 15 °C min^−1^ and were then maintained at the desired temperature until the weight stabilized, as judged by <1 wt% change over 100 min. Samples were then stored over silica gel before analysis. The isothermal annealing was carried out at 5 °C intervals in the temperature range 25–200 °C. Samples for NMR analysis were maintained at 40 °C for 1 h before the measurements to avoid transformation during the analysis^58^.

### Analysis of dehydration progress

The mechanism of dehydration of uncoated ACC in air under thermal treatment and ACC particles encapsulated in silica shells was investigated by replotting the weight loss curves as dehydration curves showing the fractional loss of total water (*α*) (below 400 °C) as a function of temperature using the water fraction present at the end of each isothermal period. These were then fitted to the results expected from common solid-state reaction models. For comparison, progressive dehydration of the ACC-SiO_2_ particles in solution at 25 °C was considered as a function of incubation time in solution, where *α* values were determined by TGA analysis (weight loss below 200 °C). Activation energies associated with the dehydration of ACC at different degrees of hydration, CaCO_3_.*x*H_2_O, in air were obtained by isoconversion methods based on recorded overlapping *α* values during isothermal storage present in flanking isotherms. A plot of ln(d*α*/d*t*)_*α*_ versus 1/*T*, where the value of (d*α*/d*t*)_*α*_ is determined for each isothermal dehydration event and temperature *T*, returns a straight line of gradient—*E*_a_/*R*.

### Characterization

The CaCO_3_ precipitates were characterized by infrared spectroscopy, PXRD, TGA, DSC, SEM, TEM and SSNMR. Crystal morphologies were characterized using SEM by mounting glass slides supporting the CaCO_3_ particles on SEM stubs using adhesive conducting pads and coating with Pt/Pd. Imaging was performed using a LEO 1,530 Gemini FEG-SEM operating at 3 kV or a FEI Nona NanoSEM 650. TEM was carried out using a FEI Tecnai TF20 FEGTEM fitted with an high-angle annular dark field detector and a Gatan Orius SC600A charge-coupled device camera, operating at 200 kV. Fluorescent confocal microscopy was performed using an Inverted Olympus IX-70 wide-field microscope with 100 W mercury illumination epifluorescence and differential interference contrast optics.

Individual crystal polymorphs and initial amorphous character were confirmed with infrared spectroscopy using a Perkin Elmer attenuated total reflectance-infrared. Further confirmation of polymorphs and polymorphic transitions was obtained with PXRD using a Bruker D8 Advanced diffractometer equipped with a CuKα_1_ X-ray source and internal heating stage. Samples were placed on a silicon wafer, and XRD data were collected at angles from 5° to 70° in intervals of 0.02, with a scan rate of 1° min^−1^. Polymorphic transitions were also followed using DSC (TA Instruments DSC Q200), with a heating rate of 10–25 °C min^−1^ under a nitrogen flow rate of 100 ml min^−1^. The compositions of the different ACC samples were investigated using TGA, where data were recorded using a TA Instruments STD Q600, with a heating rate of 10–25 °C min^−1^ under a 100-ml min^−1^ N_2_ flow. SSNMR experiments were performed using standard methodologies with a Bruker 9.4 Tesla Avance-400 wide bore spectrometer, at frequencies of 400.1 MHz (^1^H). One-dimensional data sets were acquired on samples spun at 10 kHz using MAS (^1^H π/2 pulse length 2.5 μs, contact time 2.5 ms, at a ^1^H field strength of 100 kHz) and a repetition time of 2 s was employed in all experiments. On occasions where there was insufficient material to fill a 4-mm outer diameter rotor, the unfilled volume was taken up with polytetrafluoroethylene tape. The number of scans acquired depended on the quantity of available sample, and was generally between 256 and 512.

## Author contributions

J.I. performed experiments, analysed data and wrote the paper; W.C.W. and M.J.D. collected and interpreted SSNMR spectra. E.H.N., A.N.K. and Y.-Y.K. performed preliminary experiments. H.K.C. gave conceptual advice and wrote the paper. F.C.M. supervised the project and wrote the manuscript.

## Additional information

**How to cite this article:** Ihli, J. *et al*. Dehydration and crystallization of amorphous calcium carbonate in solution and in air. *Nat. Commun.* 5:3169 doi: 10.1038/ncomms4169 (2014).

## Supplementary Material

Supplementary Information Supplementary Figures 1-5 and Supplementary Table 1

## Figures and Tables

**Table 1:** **Summary of data derived from the thermal analysis of uncoated ACC in air.**

	**~Wt% range**	***α***	**CaCO**_**3**_: ***x*****H**_**2**_**O**	***E***_**a**_ **(kJ mol**^**−1**^**)**	**Dehydration model**			
					**Type**	**f(*****α*****)=*****k*****T**	***R***^**2**^	***k*** **(°C**^**−1**^**)**
25–40 °C	20 to 14	0.0–0.3	~1.4–0.98	80	Second-order reaction	[1/(1−*α*)]−1	0.98	0.153*
40–140 °C	14 to 3	0.3–0.85	~0.98–0.25	140	Contracting volume	1−(1−*α*)^1/3^	0.92	0.0042
140–260 °C	3 to 0.5	0.85–0.95	~0.25–0.08	245	Second-order nucleation	*α*^1/2^	0.85	0.0031
25–260 °C	*−*	*−*	*−*	125	Contracting volume	1−(1−*α*)^1/3^	0.92	0.0038

*α* is the degree of dehydration, *E*_a_ is the average activation energy associated with water loss for a given temperature range and CaCO_3_: *x*H_2_O shows the number of moles of water associated with 1 CaCO_3_ formula unit. The best-fit solid-state reaction models (f(*α*)=*k*T) are further given together with the associated coefficient of determination *R*^2^ and the rate constant, *k*.

^*^f(*α*)=*k*[min^−1^]*t.*
