# Supplementary material for: Dehydration and crystallization of amorphous calcium carbonate in solution and in air
Source: Nat Commun. 2014 Jan 28;5:3169. doi: 10.1038/ncomms4169 (PMC4085778; doi:10.1038/ncomms4169)
Supplement: Supplementary Information — Supplementary Figures 1-5 and Supplementary Table 1 [file ncomms4169-s1.pdf]

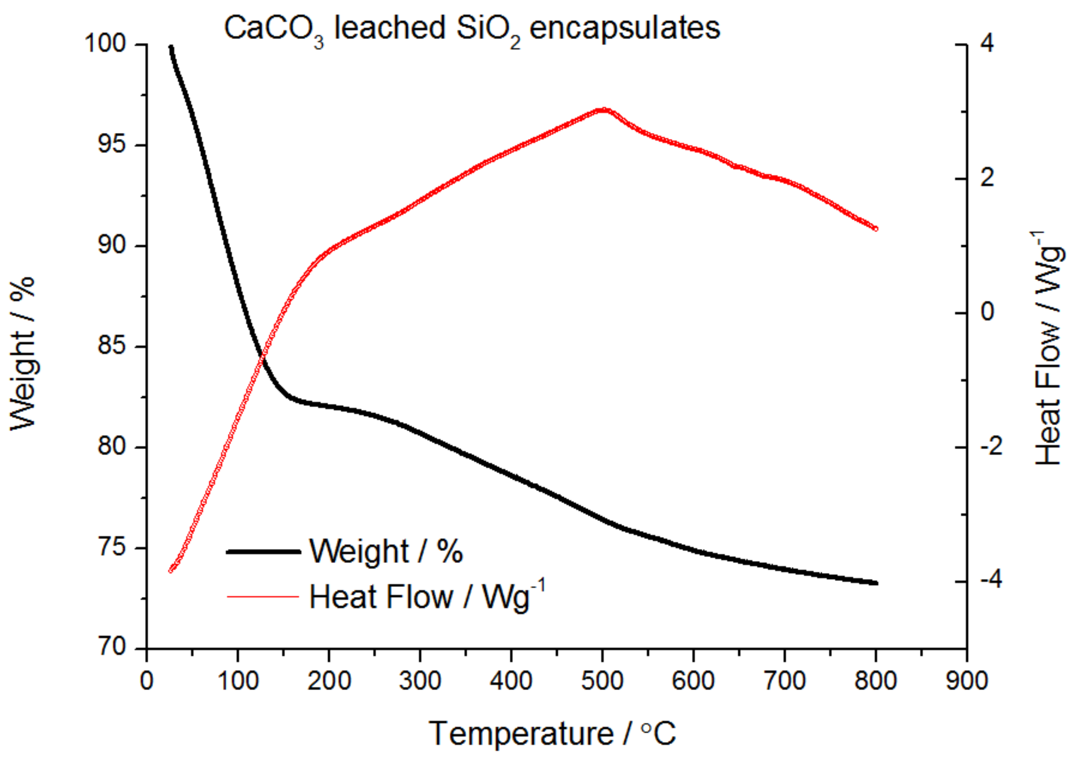


**Supplementary Figure 1 Thermogravimetric analysis (TGA) of silica shells formed by leaching the $CaCO_3$ from ACC-$SiO_2$ particles by incubating the particles in HCl for 24 hours**. TGA measurements were performed at a heating rate of 15°C /min under 100ml $N_2$/min.

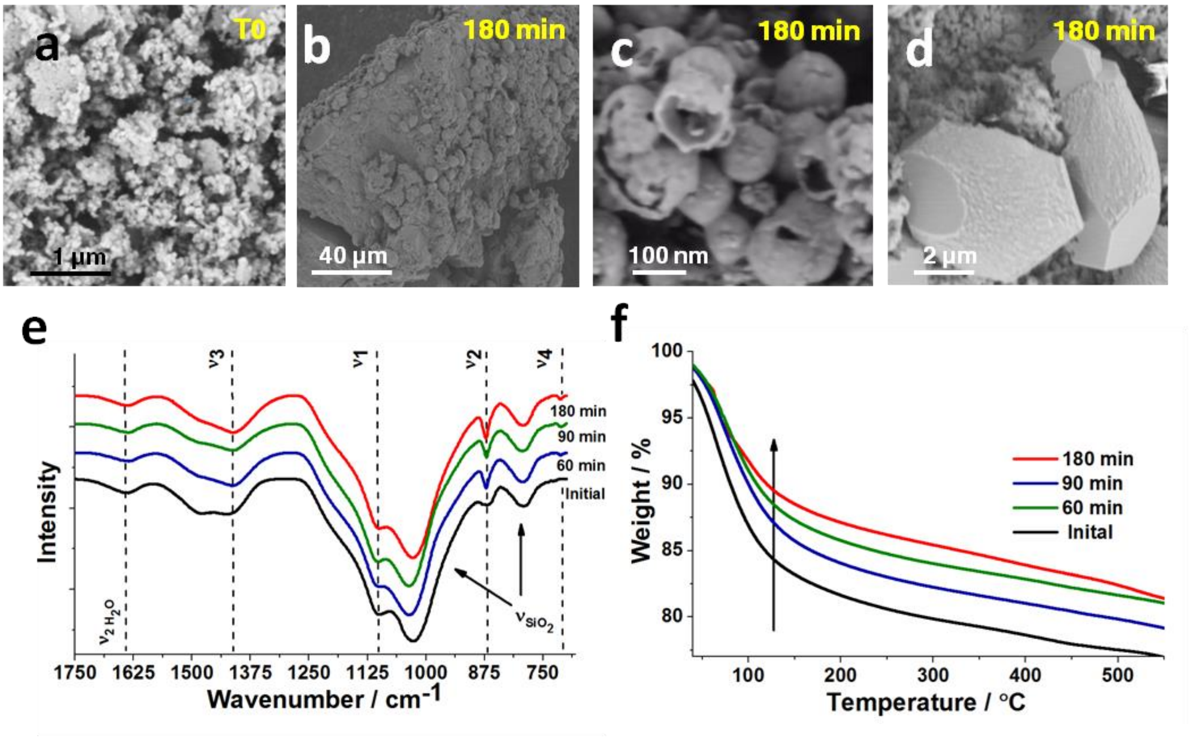


**Supplementary Figure 2 Characterization of pure ACC particles coated with silica shells and their dehydration on incubation in solution with time.** (a-d) SEM images show that the silica does not coat all ACC particles sufficiently to prevent rapid dissolution and transform to calcite. (a) Initially prepared particles are identical in appearance to ACC synthesised in the presence of $NaSiO_2$. (b) Silica-coated ACC samples after 180 minutes in solution, showing the presence of (c) empty silica shells and (d) calcite particles. These calcite particles give an apparent constant background in IR spectra. (e) IR spectra of coated ACC particles after different incubation times in solution, further indicate an increased $SiO_2$ presence in formed precipitate as compared with ACC coprecipitated with silica attributed to the higher concentration used.[36] (f) TGA also demonstrates similar dehydration behaviour of the silica-coated pure ACC particles as ACC particles co-precipitated with silica.

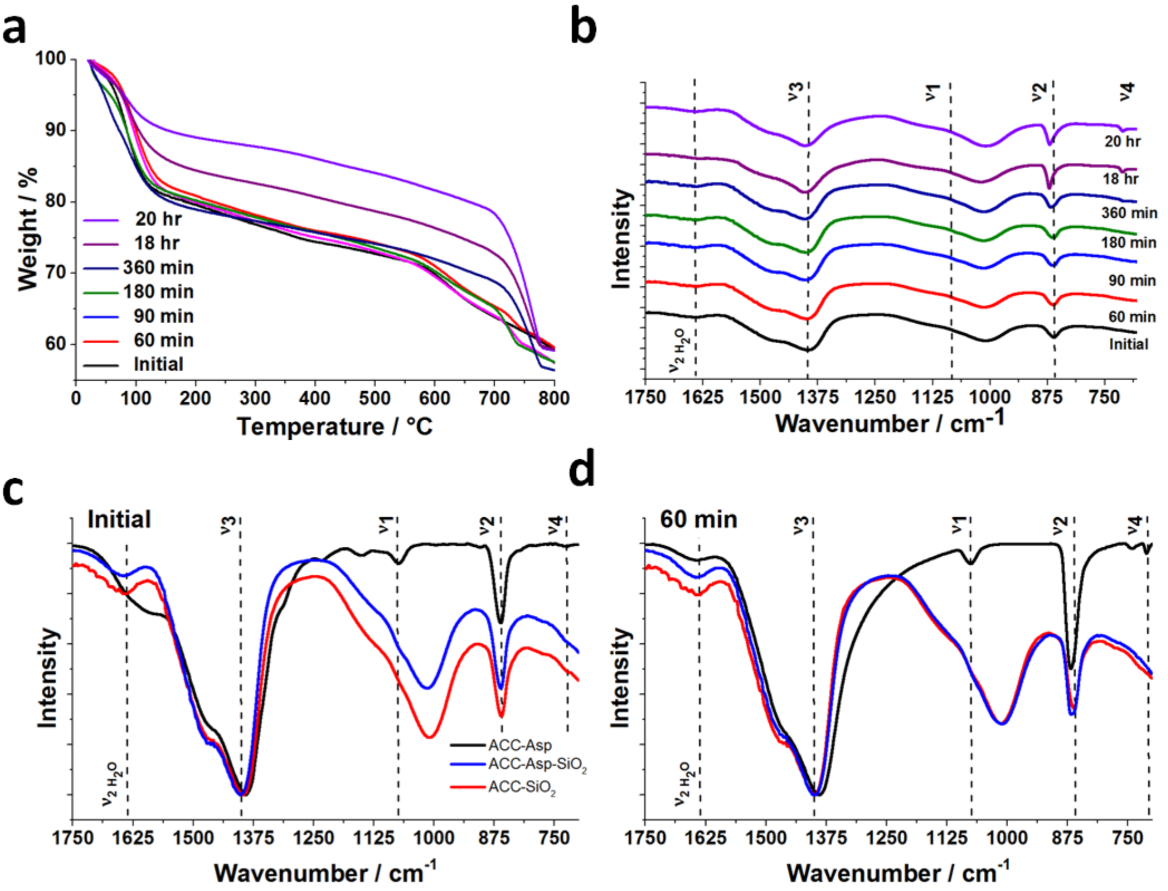


**Supplementary Figure 3 Transformation of ACC-Asp-$SiO_2$ particles prepared by combining equal volumes of 10 mM $Na_2CO_3$/ 6 mM $Na_2SiO_3$ and 10 mM $CaCl_2$ $2H_2O$/ 5 mM aspartic acid.** (a) Thermogravimetric analyses and (b) IR spectra of ACC-Asp-$SiO_2$ after different incubation times in solution. The weight loss during heating decreases with time spent in solution because progressively more dehydration has already occurred in solution. The small band at ≈ 714 $cm^{-1}$ corresponding to crystalline $CaCO_3$ was observed in IR spectra after 18 hours, with the first hint of crystallinity appearing after ≈ 5 hours. (c and d) Comparison of IR spectra of ACC-$SiO_2$ (–), ACC-Asp (–) and ACC-Asp-$SiO_2$ (–) particles after incubation in solution for (c) 0 mins and (d) 60 mins. The spectra show the development of bands for vaterite and calcite at ≈ 714 $cm^{-1}$ and 747 $cm^{-1}$ for the ACC-Asp particles after 60 mins, while these are not observed for the coated ACC particles.

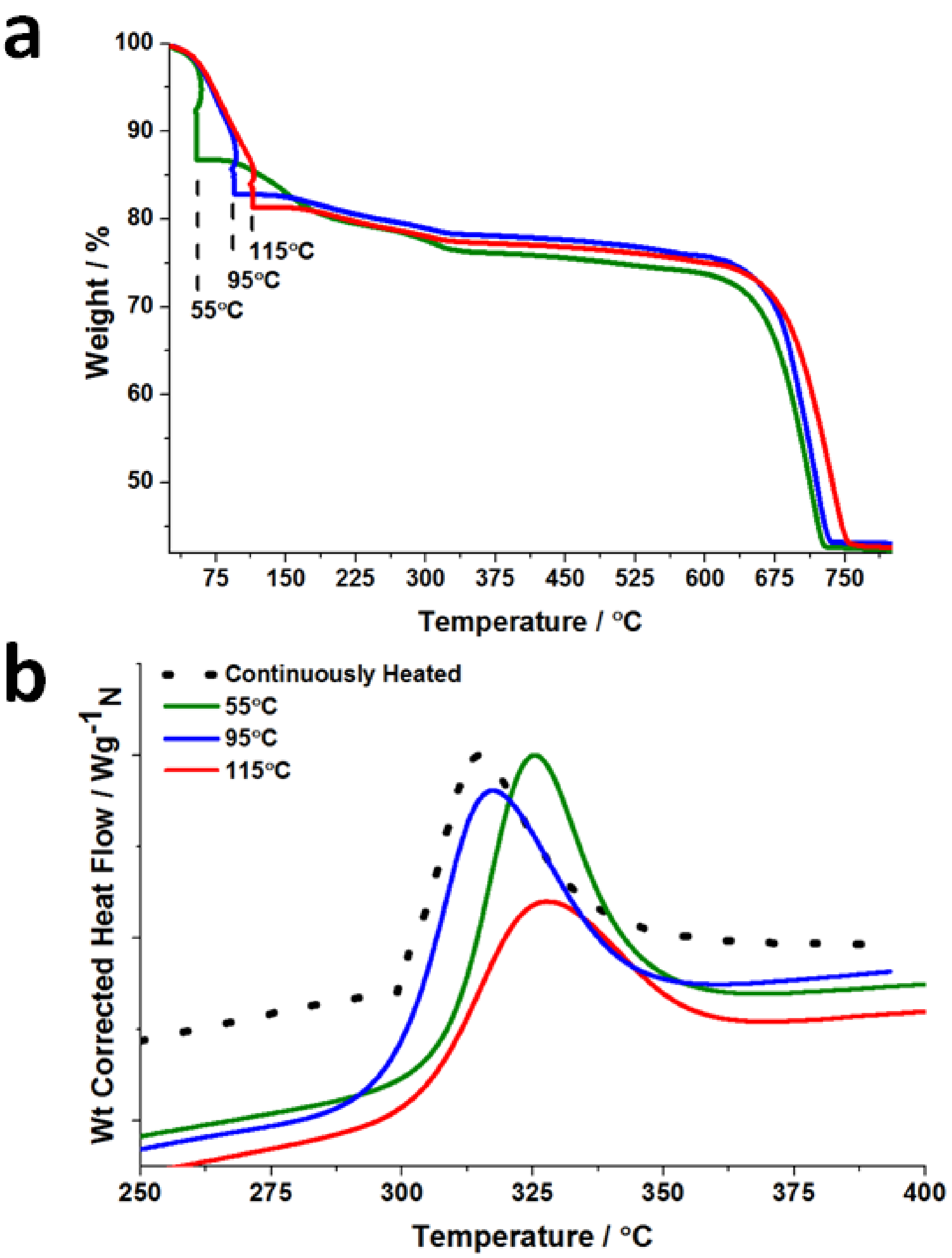


**Supplementary Figure 4 Full temperature range TGA (a) and limited DSC (b) of uncoated ACC particles.** ACC particles were produced by combining equal volumes (0.5-1.5 ml) of 1 M $(NH_4)_2CO_3$ (pH 9.15) with 1 M $CaCl_2$ (pH ~6.8) at 4$^{o}$C. A heating rate of 15°C/min with a single isothermal annealing period (for 100min at 55, 95 or 115°C) under a constant gas flow rate of 100ml $N_2$/min was used. Note from (b) that no consistent variation in the position of the crystallization peak was found.

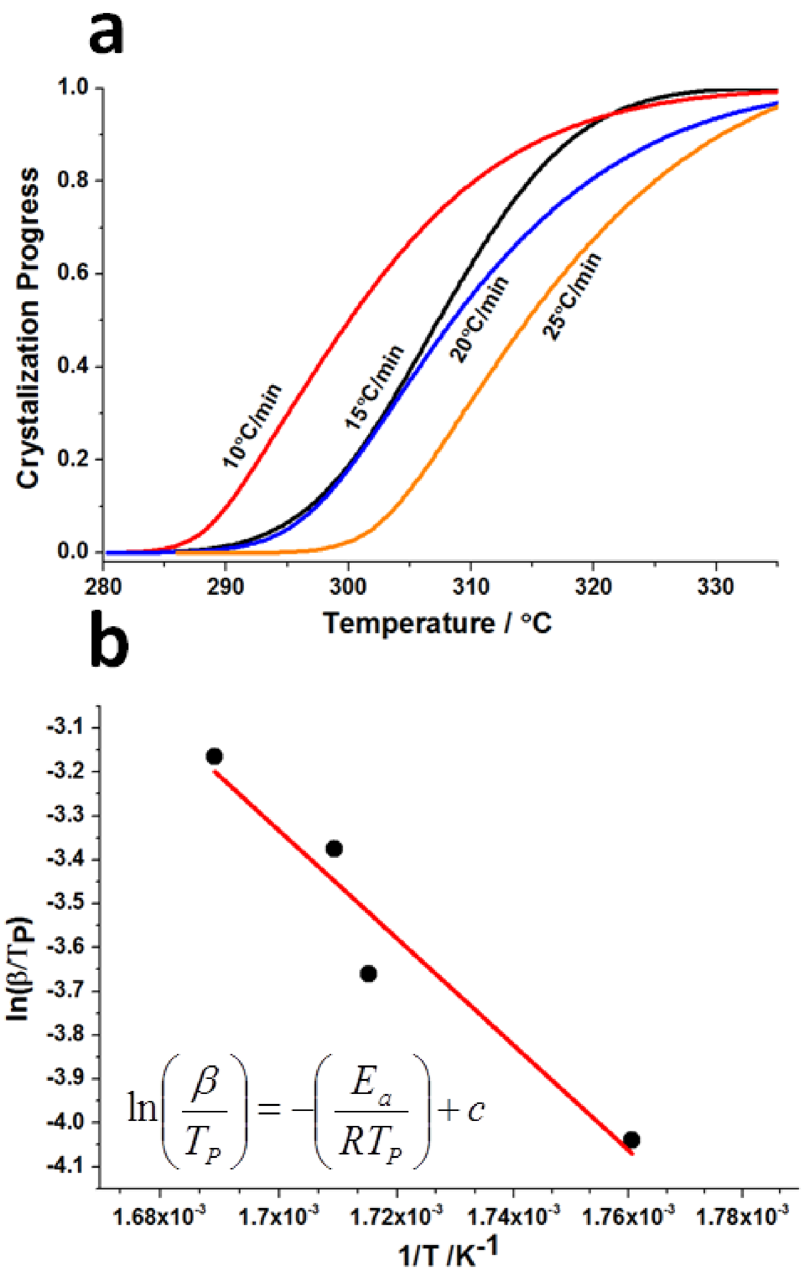


**Supplementary Figure 5 Evaluation of the Progress of ACC Crystallization.** (a) Curves showing the crystallisation progress of uncoated ACC samples under heating with an intermediate annealing period (135°C for 100min), as derived from DSC scans performed with applied heating rates of 10, 15, 20 and 25$^{o}$C/min, under 100ml $N_2$/min. The progress of crystallisation, 0-1, was obtained by integration of the crystallisation associated exothermic peak present in obtained DSC scan. (b) Corresponding Boswell plot[44] required for the calculation of activation energies associated with crystallisation, $E_a$. β = heating rate, $T_P$ = DSC central peak temperature.

**Supplementary Table 1 Summary of the intensities of FTIR peaks of uncoated ACC and ACC-$SiO_2$ particles at different stages of dehydration with respect to temperature or time.** $\alpha$ represents the degree of dehydration from 0-1, $CaCO_3$: x $H_2O$ the assumed molecular composition and Wt% $H_2O$ the total sample, water weight percentage. Spectra were normalized with respect to the right hand side of the $v_3$ peak at 1402 $cm^{-1}$ ($v_{3R}$).

**ACC after isothermal annealing**

| $\alpha$ | $CaCO_3$: x $H_2O$ | Wt% $H_2O$ | Temperature [°C] | $v_3/v_{2H2O}$ | $v_3/v_{3R}$ | $v_3/v_1$ | $v_3/v_2$ | $v_3/v_4$ | $v_3/v_{SiO2}$ |
|---|---|---|---|---|---|---|---|---|---|
| 0.00 | 1.40 | 20.00 | 25 | 6.67 | 1.22 | 1.69 | 2.78 | 46.54 | - |
| 0.49 | 0.71 | 10.20 | 50 | 9.09 | 1.20 | 1.82 | 2.50 | 50.76 | - |
| 0.78 | 0.31 | 4.40 | 100 | 10.00 | 1.18 | 2.50 | 2.33 | 47.65 | - |
| 0.98 | 0.03 | 0.40 | 180 | 11.11 | 1.32 | 3.75 | 2.08 | 49.87 | - |
| 0.99 | 0.01 | 0.20 | 200 | 14.29 | 1.20 | 4.52 | 2.00 | 37.50 | - |
| 1.00 | 0.00 | 0.00 | 250 | 16.67 | 1.23 | 4.52 | 2.00 | 11.11 | - |

**ACC-$SiO_2$ in solution**

| $\alpha$ | $CaCO_3$: x $H_2O$ | Wt% $H_2O$ | Time [min] | $v_3/v_{2H2O}$ | $v_3/ v_{3R}$ | $v_3/v_1$ | $v_3/v_2$ | $v_3/v_4$ | $v_3/v_{SiO2}$ |
|---|---|---|---|---|---|---|---|---|---|
| 0.00 | 1.40 | 20.00 | 0 | 11.29 | 1.30 | 2.50 | 2.94 | 45.33 | 1.53 |
| 0.21 | 1.11 | 15.80 | 30 | 11.11 | 1.33 | 2.63 | 2.70 | 50.00 | 1.59 |
| 0.39 | 0.85 | 12.20 | 60 | 11.11 | 1.28 | 2.78 | 2.38 | 43.47 | 1.64 |
| 0.54 | 0.64 | 9.20 | 90 | 12.50 | 1.37 | 2.78 | 2.04 | 51.37 | 1.75 |
| 0.67 | 0.46 | 6.60 | 120 | 12.50 | 1.41 | 2.94 | 1.82 | 50.98 | 1.86 |
| 0.76 | 0.34 | 4.80 | 150 | 16.67 | 1.37 | 5.00 | 1.85 | 36.33 | 2.28 |
| 0.98 | 0.03 | 0.40 | 500 | 25.00 | 1.30 | 5.26 | 1.85 | 12.50 | 2.38 |
